# Characterization of extended-spectrum cephalosporin-resistant *Klebsiella* recovered from dairy manure in Southern Ontario, Canada

**DOI:** 10.1371/journal.pone.0336012

**Published:** 2026-01-09

**Authors:** Rebecca E. V. Anderson, Gabhan Chalmers, Andrew Scott, Roger Murray, Michael Mulvey, Edward Topp, Patrick Boerlin, Nicole Ricker

**Affiliations:** 1 Department of Pathobiology, Ontario Veterinary College, University of Guelph, Guelph, Ontario, Canada; 2 Agriculture and AgriFood Canada, London, Ontario, Canada; 3 National Microbiology Laboratory, Public Health Agency of Canada, Winnipeg, Manitoba, Canada; 4 INRAE, University of Burgundy, Dijon, France; 5 Department of Biology, University of Western Ontario, London, Ontario, Canada; Yale University School of Medicine, UNITED STATES OF AMERICA

## Abstract

Extended-spectrum cephalosporin (ESC)-resistant *Klebsiella pneumoniae* are a problem in human patients and have been studied extensively. However, there is a paucity of information regarding ESC-resistant *K. pneumoniae* from livestock in general, and in Canada in particular. This study characterized ESC-resistant *K. pneumoniae* recovered from dairy manure in Ontario, Canada, and their ESC-resistance plasmids. ESC-resistant *K. pneumoniae* (*n* = 73) and *K. quasipneumoniae* (n = 11) isolates were screened by PCR for *bla*_CTX-M_, *bla*_CMY_ and *bla*_SHV_ prior to undergoing antimicrobial susceptibility testing using disk diffusion. Isolates from dairy manure carrying *bla*_CTX-M_ (*n* = 74), and additional isolates from turkeys (*n* = 8) and dogs (*n* = 2), underwent short-read whole genome sequencing (WGS) and a subset of these (*n* = 35) had additional long-read sequencing and hybrid assembly for confirmation. Isolates were characterized using multi-locus sequence typing (MLST) and antimicrobial resistance (AMR) gene profiles. Thirty known sequence types (STs) and four novel STs were identified for *K. pneumoniae,* and two STs among *K. quasipneumoniae*. The isolates were found at various treatment stages of the manure on one farm but were only sporadically found on any of the other farms investigated. The majority of isolates (86%) were multi-drug resistant. Variants of CTX-M were identified in diverse STs and included *bla*_CTX-M-15_ (*n* = 81), *bla*_CTX-M-1_ (*n* = 2) and *bla*_CTX-M-32_ (*n* = 1). The *bla*_CTX-M-15_ gene was located on diverse IncF, IncHI1 or IncY replicons as well as on the chromosome, whereas *bla*_CTX-M-1_ was harboured on the epidemic IncI1/ST3 plasmid, and *bla*_CTX-M-32_ on an IncN plasmid. Plasmids were characterized based on core gene SNPs, replicon types and AMR genes, and compared to plasmids from *Escherichia coli* from a parallel study. Transfer of *bla*_CTX-M_ plasmids between bacterial species by conjugation was also assessed. Conjugation of IncI1 and IncFII plasmids occurred from *K. pneumoniae* to *E. coli* strains but not *vice versa*. Notably, IncY replicons were identified as conjugative plasmids and transfer was demonstrated between *E. coli* strains as well as from *K. pneumoniae* to *E. coli*. No strain overlap was observed between dairy manure isolates and those from turkey and dogs, however we did identify similarities between *K. pneumoniae* MLST and resistance profiles from publicly available human clinical isolates to our isolates found in dairy manure (ST37 and 405), turkeys (ST45) and dogs (ST711).

## Introduction

*Klebsiella pneumoniae* is a member of the ESKAPE group of priority pathogens [1] and is of clinical relevance for both humans and animals. In humans, *K. pneumoniae* is a common cause of urinary tract infections, septicemia, and abdominal infections [2,3] and is amongst the top five causes of bacterial pneumonia in Canadian hospitals [4]. In animals, *K. pneumoniae* is associated with mastitis in dairy cattle, urinary tract infections (UTIs) in dogs, pneumonia in horses, and a variety of other opportunistic infections [5–7]. Farm animals such as dairy cattle may also play a role in the dissemination of enteric organisms into the environment and subsequently into the food chain through manure treatment onto crops, such as has been documented for *E. coli* transfer to lettuce [8].

Antimicrobial resistance (AMR) is a rising global health issue in humans and animals, with resistance to the critically important extended-spectrum cephalosporins (ESCs) documented originally in human clinical cases, and subsequently in companion animals and livestock [9]. More research is needed to identify the role *K. pneumoniae* plays in the spread of AMR especially since, in recent years, the frequency of multi-drug resistant (MDR) *K. pneumoniae* strains, including ESC-resistance, observed among human clinical infections has increased [1,10]. However, most research surrounding ESC-resistance in non-human sources has focused on *Escherichia coli* and *Salmonella enterica*. Ceftiofur, an ESC, is an important antimicrobial in the dairy industry for the treatment of severe clinical mastitis caused by *Klebsiella* spp. [11], particularly if the strain is resistant to first line antimicrobials [12]. Despite these concerns, there is little information regarding ESC-R *K. pneumoniae* from animals and the environment, including in Canada.

Recent studies have shown that ESC-resistance in *K. pneumoniae* is usually associated with *bla*_CTX-M_, *bla*_CMY_ and the intrinsic, *bla*_SHV_ [10,13,14] genes. The most frequent variants of CTX-M in *K. pneumoniae* from human and animal sources are CTX-M-14, −15 and −55 [15–19]. *K. pneumoniae* may therefore have a role in the dissemination of AMR genes through horizontal gene transfer (HGT) [20,21], as CTX-M-14 and −15 have been associated with conjugative plasmids in diverse hosts within the Enterobacterales [22].

Farm animals play a role in the One Health cycle due to their intimate interactions with humans (both farm workers and consumers) and the environment through manure application. The overarching goal of this study was to gain insights into the role that *K. pneumoniae* plays in the context of antimicrobial resistance and One Health in relation to dairy cattle. The objectives of this study were threefold. The first was to assess the diversity and distribution of ESC-resistant *K. pneumoniae* in a sample of dairy farms in Ontario. The second objective was to characterize the diversity and distribution of *bla*_CTX-M_-harbouring plasmids within the dairy manure collection compared to other relevant sources, including other animal species in our collection and sequenced plasmids available through public databases. Finally, the last objective was to understand the transferability of *bla*_CTX-M_-harbouring plasmids between *K. pneumoniae* and *E. coli*. The results of the study will contribute to a better understanding of the epidemiology of ESC-resistance in *K. pneumoniae* in Canada, and of transmission of ESC-resistance plasmids between bacterial species.

## Materials and methods

### Farm sampling and bacterial isolates

*K. pneumoniae* isolates (*n* = 120) were screened using polymerase chain reaction (PCR) for *bla*_CTX-M_, *bla*_CMY_, and *bla*_SHV_ (Table 1). PCR positive isolates (n = 84) were identified from 34 manure samples recovered in four of six sampled farms in Southern Ontario between November 2018 and August 2019 [23]. Manure samples were taken before treatment, during anaerobic digestion at mesophilic and thermophilic temperatures, and at the final output stages including the liquid manure applied to land under cultivation to grow crops, and the dewatered material used for livestock bedding [23]. Additionally, ESC-resistant *K. pneumoniae* from turkey fecal samples (*n *= 23) [24] and dog clinical samples (*n* = 13) [25] were also investigated. Species identification was performed using matrix-assisted laser desorption ionization time of flight (MALDI-TOF; Bruker Daltonik GmbH, Bremen, Germany) at the Animal Health Laboratory (AHL), University of Guelph, ON.

**Table 1:** Primers used for PCR screening to characterize ESC-resistant isolates.

Target	Primer Sequence	Product size (bp)	Reference
*bla*_CTX-M_	F: ATGTGCAGYACCAGTAARGTKATGGC R: TGGGTRAARTARGTSACCAGAAYCAGCGG	593	[26] Mulvey et al., 2003
*bla*_CMY_	F: GACAGCCTCTTTCTCCACA R: TGGACACGAAGGCTACGTA	1000	[27] Kozak et al., 2009
*bla*_SHV_	F: AGGATTGACTGCCTTTTTG R: ATTTGCTGATTTCGCTCG	393	[27] Kozak et al., 2009

### Characterization of ESC-resistant *K. pneumoniae*

Antimicrobial susceptibility profiles were determined using the disk diffusion method, following guidelines and interpretation criteria from CLSI [28,29] for cefoxitin (FOX), cefotaxime (CTX), ceftazidime (CAZ), ertapenem (ETP), gentamicin (GEN), ampicillin (AMP), amoxicillin-clavulanic acid (AMC), sulphonamide compounds (SUL), sulfamethoxazole-trimethoprim (SXT), tetracycline (TET), streptomycin (STR), kanamycin (KAN), ciprofloxacin (CIP) and chloramphenicol (CHL). Isolates resistant to antimicrobials from three or more antimicrobial classes, not including intermediate phenotypes, were classified as MDR. For the sake of defining MDR, the ß -lactamase category was divided into the following sub-categories: cephems (CTX, CAZ and FOX), ß-lactam/ß-lactamase inhibitor combinations (AMC), carbapenems (ETP) and other ß-lactams (AMP). AMC was not considered in the determination of multi-drug resistance since carrying *bla*_CMY_ would naturally result in multi-drug resistance (i.e., resistance to cephems, other ß-lactams, and ß-lactam/ß-lactamase inhibitor combinations).

### Whole genome sequencing (WGS)

*K. pneumoniae* carrying *bla*_CTX-M_ from dairy manure (*n* = 74), turkey (*n* = 8) and dog (*n* = 2) underwent short read WGS. Preparation of DNA for WGS was done using Epicentre MasterPure^TM^ DNA Purification kit (Epicentre, Madison, WI) following the manufacturer’s instructions. Sequencing was done either with Illumina Miseq (PE300; Illumina, San Diego, CA) at the Advanced Analysis Centre, University of Guelph, Guelph, ON, or Illumina NextSeq (PE150) at the National Microbiology Laboratory in Winnipeg, MB, (NML Winnipeg).

A subset of isolates carrying *bla*_CTX-M_ from dairy manure (*n *= 27), in addition to the isolates from turkey feces (*n* = 8) and dog clinical samples (*n* = 2), were also sequenced using the MinION Platform (Oxford Nanopore Technologies). The subset of dairy manure isolates were selected using a phenetic tree created with the inhibition zone diameters from antimicrobial susceptibility testing. This tree was created in BioNumerics v7.6 (Applied Maths, Austin TX. USA) using Pearson correlation coefficients and cluster analysis with the unweighted pair group method with arithmetic mean. One or two isolates were selected from each cluster defined at a 94% cut off, to include the highest diversity of strains from different time periods and treatment processes. Preparation of DNA samples was performed as above. Sequencing libraries and barcoding preparation were done using the SQK-LSK109 and EXP-NBD104/114 ligation and native barcoding kits (Oxford Nanopore Technologies) according to the manufacturer’s instructions. Flow cells (version FLO-MIN106 R9.4) were run for 72h each, or until exhausted. Basecalling of fast5 files and demultiplexing was performed using Guppy Basecaller v3.3 (Oxford Nanopore Technologies) with barcode trimming enabled.

### Sequence analysis

Short reads were first used to assemble the genome sequences in BioNumerics v7.6 (Applied Maths, Austin TX. USA) using the SPAdes *K. pneumoniae de novo* assembler, and assembly-free and assembly-based allele calling. Resistance genes and plasmid markers were identified using the *E. coli* functional genotype plug-in for BioNumerics at a 90% similarity which utilizes ResFinder and PlasmidFinder databases from the Center for Genomic Epidemiology (Technical University of Denmark, DTU). Multi-locus sequence types (MLSTs) were manually assigned using a MLST finder database also from the Center for Genomic Epidemiology [30,31] with paired end Illumina reads. Any isolates with an unknown sequence type (ST) due to a novel allele or novel allelic configuration, were thoroughly analyzed to identify any errors in allelic regions by reference mapping of short reads. If no errors were present, and no ST was assigned, these strains were submitted to the Pasteur Institute (https://bigsdb.pasteur.fr/klebsiella/) and assigned novel ST designations.

### Plasmid assembly and analysis

Long and short reads were assembled using hybrid assembler Unicycler v0.4.8 [32], in parallel with Flye v2.6 [33] and were polished with short reads using Racon v1.4.0 [34] and Pilon v1.23 [35]. Both Flye and Unicycler pipelines were used for quality control purposes; however, Unicycler assemblies were used for all downstream analysis. Assemblies were visualized using Bandage v0.8.1 [36] and annotated with ABRicate v0.9.8 [37] against the ResFinder v4.1 [38], PlasmidFinder v2.1 [39] and VFDB [40] databases. Sequenced genomes were analyzed using Geneious v9.1.8 (Biomatters, Auckland, New Zealand) and plasmids were aligned and visualized using Mauve plug-in v2.3.1 [41]. Alignment of plasmids was performed using EasyFig [42].

### Analysis of genetic relationships

Minimum spanning trees (MSTs) were generated using the whole genome multi-locus sequence typing (wgMLST) (core enterobase) function for core genome MLST (cgMLST) trees in BioNumerics v7.6 (Applied Maths, Sint-Martens-Latem, Belgium) with 10x bootstrapping and no multithreading. This approach utilized the *Klebsiella pneumoniae* WGS scheme, which includes 634 core loci.

Core gene phylogenetic analyses on isolates recovered from dairy manure were also performed using Snippy v4.4.5 according to developer’s guidelines, under Core SNP Phylogeny (https://github.com/tseemann/snippy) with *K. pneumoniae* type strain ATCC 13883 (GenBank Accession #JOOW00000000.1) and *K. quasipneumoniae* subsp. *quasipneumoniae* strain 01A030 (GenBank Accession #GCA_000751755.1) as reference strains. The clean.full.aln files were analyzed with Gubbins v2.4.0 [43] and the output clean.core.aln file was analyzed with SNP-sites v3.0 [44]. FastTree v2.2.11 [45] was used to generate the trees which were visualised in Geneious v9.1.8.

In order to compare *bla*_CTX-M_ harbouring plasmids, phylogenetic SNP analyses of the hybrid assembled ESC-resistance plasmids were conducted using a pre-determined protocol [46]. A gene presence/absence analysis was first conducted on all ESC-resistance plasmids independently of replicon types, including the ESC-plasmids in *Klebsiella spp*. from dairy cattle and turkey, in addition to ESC-resistance plasmids in *E. coli* from the same dairy manure samples [23]. These plasmids (*n* = 65) were first analyzed with Prokka v1.14.6 (https://github.com/tseemann/prokka) with the output.gff files analyzed using Roary v3.13.0 [47] A SNP analysis was then performed individually on IncY replicon, IncI1 and IncFII plasmids. Analysis for both IncY replicons and IncI1 plasmids also incorporated plasmids from *E. coli* isolated in a parallel study [23] using the SNP-sites generated from the core.gene.alignment output file from Roary. The SNP sites along with the core.gene.alignment file, were used to create a maximum likelihood tree with IQtree v2.0.3 [48]. All trees were visualized in Geneious v9.1.8 (Biomatters, Auckland, New Zealand).

### Conjugation assays

To determine inter-species transfer of *bla*_CTX-M_ harbouring plasmids through conjugation, *K. pneumoniae* (*n* = 3) were used as donors and *E. coli* CV601*gfp*Kan^R^Rif^R^ as recipient strain [49]. Transfer of *bla*_CTX-M_ plasmids from *E. coli* donors (*n* = 3) to the *K. pneumoniae* ATCC13883∆Nal^R^ recipient was also assessed. *K. pneumoniae* and *E. coli* donors included isolates which carried either IncI1 or IncF plasmids from previous studies [23].

All conjugation assays were quantitative and conducted in biological triplicate at 30˚C and 37˚C. For broth conjugations, cultures were grown overnight at 37˚C with shaking in 5 ml LB broth containing appropriate selective antimicrobials for donor *E. coli* and *K. pneumoniae* strains (CTX 2 mg/ml), recipient *E. coli* (RIF 50 mg/ml and KAN 50 mg/ml) and recipient *K. pneumoniae* (NAL 64 mg/ml) for the donor and recipient strains. Cultures were pelleted, washed three times with LB broth containing no antibiotics and resuspended. Donor (200µl) and recipient (200µl) strains were added to 1.6 ml LB broth and incubated for 16h without shaking at 30˚C or 37˚C. Conjugations were serially diluted and plated (100µl from each dilution) on appropriate selective and non-selective media. Three colonies per transconjugant selective medium were grown in pure cultures and confirmed to carry *bla*_CTX-M_ using PCR. Final conjugation populations were calculated based on the ratio of transconjugants to recipient CFU/mL [49]. In addition to the broth mating for *E. coli* donors to *K. pneumoniae* recipients, surface mating was also conducted. Donor (20µl) and recipient (20µl) strains were inoculated onto filter paper (1 cm^2^) on LB agar and incubated for 8h at 30˚C or 37˚C. Filter papers were removed with sterile forceps and added to 1 ml of LB broth, vortexed and serially diluted on appropriate selective media.

The transferability of *bla*_*CTX-M-15*_*-*harbouring IncY plasmids was assessed through quantitative filter-mating conjugation experiments performed in biological triplicate and technical duplicate. *E. coli* isolate 343-3b [23], and *K. pneumoniae* isolate 343-2b were used as donor strains, while *E. coli* CV601*gfp*Kan^R^Rif^R^ [49] was used as the recipient strain. Strains were inoculated into 20 mL LB broth supplemented with cefotaxime (2 mg/L) for donor strains and rifampicin (16 mg/L) for the recipient strain. Filter plating was performed as described above, except that 100 μL of donor and recipient were plated and conjugation was allowed to continue for 24 hours before plating. Two independent transconjugant colonies (one circular, and one punctiform) from the *K. pneumoniae* to *E. coli* and the *E. coli* to *E. coli* conjugations were extracted using the MasterPure™ Complete DNA and RNA Purification Kit (Lucigen, cat no MC85200) before long read sequencing was performed using a MinION Mk1B device (Oxford Nanopore Technologies, Oxford, United Kingdom).

## Results

### Antimicrobial susceptibility and multi-drug resistance

*Klebsiella spp.* carrying *bla*_CTX-M,_ *bla*_CMY_ or *bla*_SHV_ (*n* = 84) from dairy cattle were identified through PCR screening from multiple stages of manure treatment across four Ontario dairy farms. All isolates were primarily identified as *K. pneumoniae* using MALDI-TOF. However, further analysis using full length 16S rRNA gene read mapping identified eleven isolates as *Klebsiella quasipneumoniae* subspecies *quasipneumoniae,* all of which carried *bla*_OKP_ instead of the intrinsic *bla*_SHV_ gene, as identified through WGS. The *K. pneumoniae* and *K. quasipneumoniae* will be described accordingly throughout this study. Only two farms (n = 5 isolates) were positive for *Klebsiella* spp. carrying *bla*_CMY_. Eighty-three percent (70/84) of the *Klebsiella* spp. isolates from dairy manure carrying *bla*_CTX-M_ and *bla*_SHV_ or *bla*_OKP_ were multi-drug resistant (MDR) (Table 2). As expected, all isolates from dairy cattle carrying *bla*_CTX-M_ (77 total) were resistant to ampicillin and cefotaxime, while 90.9% (70/77) were resistant to ceftazidime and 50.6% (39/77) had an intermediate phenotype for amoxicillin-clavulanic acid. Only one isolate (1.3%; 1/77) was fully resistant to amoxicillin-clavulanic acid and it did not carry any additional ß-lactamase genes. Among *Klebsiella spp*. carrying *bla*_CTX-M_, no resistances to ertapenem or cefoxitin were observed, and the most frequent resistances other than to ß-lactams were to tetracyclines (77.9%; 60/77), streptomycin (64.9%; 50/77), sulfonamide (57.1%; 44/77) and sulfamethoxazole-trimethoprim (54.5%; 42/77). The least common resistances were to chloramphenicol (9.1%; 7/77), kanamycin (6.5%; 5/77) and gentamicin (2.6%; 2/77). Although 27.3% (21/77) of the isolates had an intermediate susceptibility phenotype for ciprofloxacin, none were fully resistant. All *bla*_CTX-M_-positive isolates were subjected to WGS; however, three were removed from further analysis due to low sequencing coverage, leaving 63 *K. pneumoniae* and 11 *K. quasipneumoniae* in the WGS analysis. Isolates carrying *bla*_CMY_ from dairy manure and isolates solely carrying *bla*_SHV_ underwent susceptibility testing to the panel of antimicrobials but were not included in WGS. Among these, most isolates carrying *bla*_CMY_ with *bla*_SHV_ (3/5) and all isolates carrying *bla*_SHV_ only (2/2), were MDR. Among *K. pneumoniae* from turkey feces (n = 23), all isolates carrying *bla*_CTX-M_, *bla*_CMY_, or *bla*_SHV_ were MDR (Table 2). Similarly, isolates from dog clinical samples (n = 13) were all MDR except for one isolate carrying *bla*_CMY_ with no detectable *bla*_SHV_ (Table 2).

**Table 2:** Antimicrobial susceptibility profiles for ESC-resistant *Klebsiella spp*. Recovered from dairy manure, turkey feces and dog clinical samples. Antimicrobial susceptibility profiles were based on disk diffusion using cefoxitin (FOX), cefotaxime (CTX), ceftazidime (CAZ), ertapenem (ETP), gentamicin (GEN), ampicillin (AMP), amoxicillin-clavulanic acid (AMC), sulfonamide (SUL), sulfamethoxazole-trimethoprim (SXT), tetracycline (TET), streptomycin (STR), kanamycin (KAN), ciprofloxacin (CIP) and chloramphenicol (CHL). Antimicrobials were grouped to determine multi-drug resistance; aminoglycosides (KAN, GEN, STR), tetracyclines (TET), quinolones (CIP), folate pathway inhibitors (SUL, SXT), phenicols (CHL), cephems (CTX, CAZ, FOX), carbapenems (ETP) and other ß-lactams (AMP). Intermediate was considered susceptible for the multi-drug resistance classification and AMC was excluded from multi-drug resistance classification. Abbreviations: An.: animal source; DM: dairy manure; T: turkey; D: dog; MDR: multi-drug resistant.

An.	Gene(s)	% of isolates with intermediate susceptibility or resistant																												MDR/ total isolates
		KAN		GEN		STR		TET		CIP		SUL		SXT		CHL		CTX		CAZ		FOX		AMC		ETP		AMP		
		R	I	R	I	R	I	R	I	R	I	R	I	R	I	R	I	R	I	R	I	R	I	R	I	R	I	R	I	
DM	CTX-M (SHV +)	7.5 (5)	0	3 (2)	0	60.6 (40)	3 (2)	75.7 (50)	0	0	18.1 (12)	50 (33)	0	46.9 (31)	4.5 (3)	10.6 (7)	0	100 (66)	0	89.3 (59)	9 (6)	0	0	1.5 (1)	46.9 (31)	0	0	100 (66)	0	54/66
	CTX-M (OKP +)	0	0	0	0	90.9 (10)	0	90.9 (10)	0	0	81.8 (9)	100 (11)	0	100 (11)	0	0	0	100 (11)	0	100 (11)	0	0	0	0	72.7 (8)	0	0	100 (11)	0	11/11
	CMY (SHV +)	0	0	0	0	60 (3)	0	60 (3)	0	0	0	60 (3)	0	60 (3)	0	60 (3)	0	100 (5)	0	100 (5)	0	100 (5)	0	100 (5)	0	0	0	100 (5)	0	3/5
	SHV Only	50 (1)	0	50 (1)	0	50 (1)	0	100 (2)	0	0	50 (1)	100 (2)	0	100 (2)	0	0	0	100 (2)	0	100 (2)	0	0	0	0	100 (2)	0	0	100 (2)	0	2/2
T	CTX-M (SHV +)	62.5 (5)	12.5 (1)	62.5 (5)	0	87.5 (7)	0	62.5 (5)	0	50 (4)	12.5 (1)	100 (8)	0	100 (8)	0	0	0	100 (8)	0	50 (4)	50 (4)	0	0	37.5 (3)	37.5 (8)	0	0	100 (8)	0	8/8
	CMY (SHV -)	100 (1)	0	0	0	100 (1)	0	100 (1)	0	0	0	100 (1)	0	100 (1)	0	0	0	100 (1)	0	100 (1)	0	100 (1)	0	100 (1)	0	0	0	100 (1)	0	1/1
	SHV Only	57.1 (8)	7.1 (1)	50 (7)	0	57.1 (8)	0	85.7 (12)	0	57.1 (8)	0	100 (14)	0	71.4 (10)	0	0	0	100 (14)	0	21.4 (3)	64.3 (9)	0	0	14.3 (2)	78.6 (14)	0	7.1	100 (14)	0	14/14
D	CTX-M (SHV +)	100 (2)	0	100 (2)	0	100 (2)	0	100 (2)	0	0	100 (2)	100 (2)	0	100 (2)	0	0	0	100 (2)	0	50 (1)	50 (2)	0	0	50 (1)	50 (2)	0	0	100 (2)	0	2/2
	CMY (SHV +)	50 (5)	50 (5)	100 (10)	0	100 (10)	0	100 (10)	0	80 (8)	0	100 (10)	0	90 (9)	10 (1)	90 (9)	0	90 (9)	10 (1)	100 (10)	0	100 (10)	0	100 (10)	0	0	10	100 (10)	0	10/10
	CMY (SHV -)	0	0	0	0	0	0	0	0	0	0	0	0	0	0	0	0	100 (1)	0	100 (1)	0	100 (1)	0	100 (1)	0	0	0	100 (1)	0	0/1

### ESC-resistance gene diversity

Among the three animal sources, three *bla*_CTX-M_ variants were identified using WGS which included *bla*_CTX-M-15_ (*n* = 81), *bla*_CTX-M-1_ (*n* = 2) and *bla*_CTX-M-32_ (*n* = 1). Of the 81 isolates carrying *bla*_CTX-M-15_ (70 *K. pneumoniae* and 11 *K. quasipneumoniae*), 73 also carried *bla*_TEM-1_ and eight of these also carried *bla*_OXA-1_. The isolates carrying *bla*_CTX-M-1_ or *bla*_CTX-M-32_ did not carry either *bla*_TEM-1_ or *bla*_OXA-1_ (Table 3). Most dairy manure ESC-R isolates investigated (73/84, 87%) carried a *bla*_SHV_ gene. The remaining 11 all carried *bla*_OKP_ and were shown by WGS to be *K. quasipneumoniae*. Of the *bla*_SHV_ gene variants identified, the only known extended-spectrum ß-lactamase (ESBL) was SHV-27, which was found in 4 isolates. There were also 3 novel SHV variants identified, with isolates 233-1c and 233-3c having the same variant (Table 3). These two isolates were from the same sample and have the same ST, suggesting they could be the same strain. The variants of *bla*_SHV_ found in *K. pneumoniae* from turkey feces (*bla*_SHV-201_ and *bla*_SHV-148_) and from dog samples (*bla*_SHV-187_) were not found in any *Klebsiella spp*. from dairy manure (Table 3). There were also two isolates from dairy cattle manure that had truncated *bla*_SHV_ genes.

**Table 3:** Source and characteristics of *Klebsiella* spp. isolates carrying *bla*_CTX-M_ recovered from dairy cattle manure, turkey feces or dog clinical samples in Canada. Isolates carrying *bla*_OKP_ instead of *bla*_SHV_ are *K. quasipneumoniae* subsp. *quasipneumoniae* and bolded STs are those recovered in samples from more than one farm source. SHVΔ refers to truncated SHV genes.

Animal Source	Farm	Prov.	Manure Treatment^1^	Isolate ID	MLST	CTX-M	SHV or OKP	TEM-1
Dairy manure	2	ON	Raw	327-1b	ST469	15	SHV-11	–
		ON	Digestate with solids	328-1b	ST405	15	SHV-76	Yes
		ON		267-1a	ST469	15	SHV-11	–
	3	ON	Digestate with solids	269-2e, 269-3b	**ST3369**	1	SHV-1	–
		ON	Digestate without solids	233-1c, 233-3b	ST1418	15	SHV-254	Yes
	5	ON	Raw	335-2b	ST6320	15	SHV-253	Yes
		ON	Dewatered	246-3a, 246-3b	**ST2159**	15	SHV-28	Yes
	7	ON	Raw	187-1f	ST278	15	SHV-11	Yes
		ON		187-2f	ST34	15	SHV-25	Yes
		ON		187-2g, 187-3e	ST5108	15	SHV-11	Yes
		ON		206-1a, 206-3f	ST5682	15	OKP	Yes
		ON		206-2a	ST5837	15	SHV-37	Yes
		ON		235-1d, 235-2b	ST278	15	SHV-11	Yes
		ON		235-3a	ST5682	15	OKP	Yes
		ON		257-1b	ST1966	15	SHV-27	Yes
		ON		257-2c, 257-3b	ST278	15	SHV-11	Yes
		ON		279-1b, 279-3b	ST5682	15	OKP	Yes
		ON		279-2b	ST278	15	SHV-11	Yes
		ON		301-1c, 301-2b, 301-3a	ST5682	15	OKP	Yes
		ON		301-1g	ST37	15	SHVΔ	Yes
		ON		301-3b	ST7679	15	SHV-11	Yes
		ON		340-1b, 340-2c, 340-3a	ST278	15	SHV-11	Yes
		ON		345-1b	ST706	15	SHV-25	Yes
		ON		345-2b	ST5108	15	SHV-11	Yes
		ON		345-3b	ST5682	15	OKP	Yes
		ON	Digestate with solids	188-1a	ST7678	15	SHV-255	Yes
		ON		236-1b	ST2572	15	SHV-11	Yes
		ON		236-2a	ST278	15	SHV-11	Yes
		ON		258-1b	ST945	15	SHV-1	Yes
		ON		258-1c	ST183	15	SHV-119	Yes
		ON		280-1b	**ST2159**	15	SHV-28	Yes
		ON		280-2b	ST107	15	SHV-1	Yes
		ON		280-3b	ST5682	15	OKP	Yes
		ON		302-1d	ST550	15	SHV-60	Yes
		ON		302-2b	ST219	15	SHV-1	–
		ON		302-3b, 302-3c	ST168	15	SHV-11	–
		ON		341-2b	ST286	15	SHV-168	Yes
		ON		346-1b	ST791	15	SHV-1	Yes
		ON		346-3b	**ST3369**	32	SHV-1	–
		ON	Digestate without solids	281-2c, 281-3b	ST1966	15	SHV-27	Yes
		ON		303-2b	ST1655	15	SHVΔ	Yes
		ON		342-1b	ST914	15	SHV-27	Yes
		ON		342-2b	ST7679	15	SHV-11	Yes
		ON		342-3b	ST107	15	SHV-1	Yes
		ON		347-1b, 347-2b, 347-3b	ST183	15	SHV-119	Yes
		ON	Dewatered	190-1b, 190-1c	ST1517	15	SHV-11	Yes
		ON		190-3a	ST183	15	SHV-119	Yes
		ON		209-1a	ST706	15	SHV-25	Yes
		ON		238-1c	ST163	15	SHV-11	Yes
		ON		282-2b	ST219	15	SHV-1	–
		ON		304-1b	ST5682	15	OKP	Yes
		ON		304-2b	ST219	15	SHV-1	–
		ON		343-2b	ST4263	15	SHV-11	Yes
		ON		348-2b, 348-3b	ST183	15	SHV-119	Yes
		ON	Heat treated compost	261-1a, 261-1b	ST219	15	SHV-1	Yes
Turkey	131	ON	N/A	60.3*	ST147	15	SHV-11	Yes
	165	BC	N/A	17.1, 17.3	ST1564	15	SHV-201	Yes
	145	QC	N/A	142.2*	ST215	15	SHV-148	Yes
	176	QC	N/A	69.2, 70.3, 71.2, 72.2	ST45	15	SHV-148	Yes
Dog	N/A	ON	N/A	M53, M59	ST711	15	SHV-187	Yes

^1^ Manure treatments refer to the stage in the treatment process in which the samples were obtained. Details regarding each treatment can be found in a previous study [23].

*K. pneumoniae isolates from turkey feces (142.2 and 60.3) along with the two isolates from dog urine were obtained from 2016 (*n* = 2) [24,25]. The remaining isolates from turkey feces were obtained in 2017.

Prov., Province in Canada where sample was obtained, including Ontario (ON), Quebec (QC) and British Columbia (BC); Non-applicable (N/A).

The majority of isolates carrying *bla*_CTX-M_ were from the largest farm of the study (Farm 7, 64/74 isolates). Although ESC-R isolates were found throughout the manure treatment process, recovery was consistently low and highly variable, which did not allow for delineation of any clear trends with respect to manure treatment stages. Isolates positive for *bla*_CTX-M_ were found in raw manure in three farms, in the liquid end-product to be applied to the environment in two farms and in the dried end-product to be recycled as bedding in two farms. The eleven *K. quasipneumoniae* isolates were also all from Farm seven. They were found mainly in raw manure, but also in two different downstream processing stages (Table 3).

### Sequence type diversity among *bla*_CTX-M_ positive isolates

**Fig 1:**
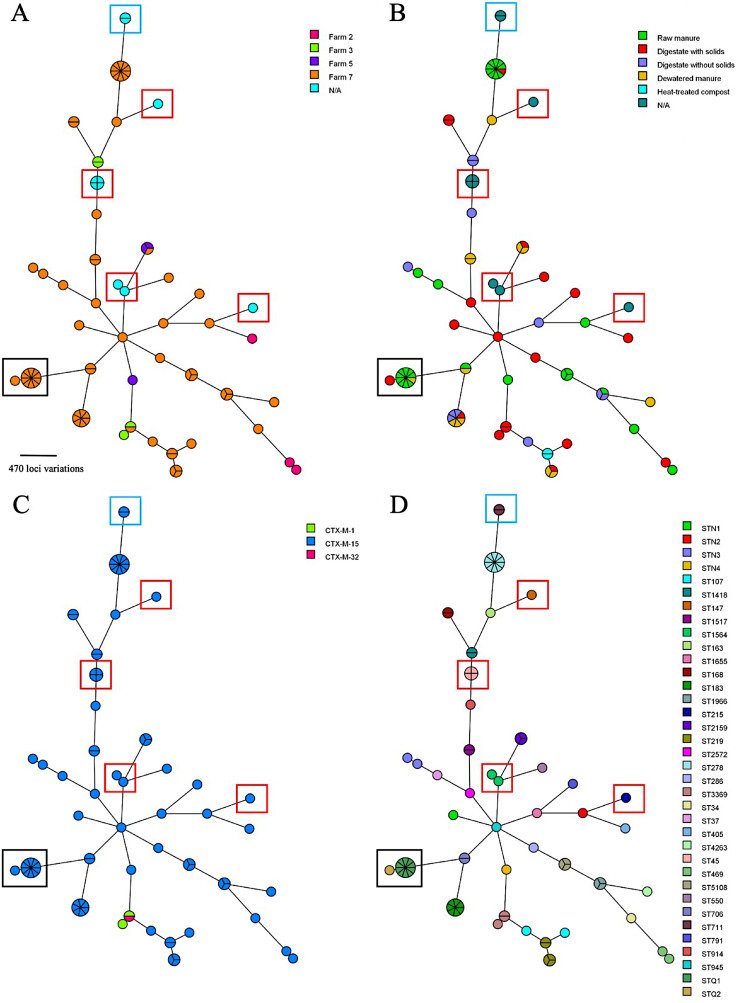
Minimum spanning trees created using cgMLST with 634 core genome loci for *K. pneumoniae* (*n* = 63) and *K. quasipneumoniae* (*n* = 11) isolates from dairy manure, turkey feces (*n* = 8) and dog urine (*n *= 2). Trees are colour coded based on A) dairy farm, B) dairy manure treatment process, C) *bla*_CTX-M_ variant and D) MLST profile. The black square encapsulates the *K. quasipneumoniae* isolates (*n* = 11), blue square encapsulates *K. pneumoniae* from dog urine samples and the red squares indicate isolates from turkey feces. On all farms, digestate with solids is the first anaerobically digested manure and digestate without solids is environmentally applied manure. On farm five, dewatered manure is used as recycled bedding whereas on farm seven, dewatered manure is thermophilically treated to create compost, which is then used as recycled bedding.

Among isolates from dairy manure, 30 STs were identified through multi-locus sequence typing (MLST) (Table 3). Additionally, five isolates were identified as belonging to four different novel STs, ST7678, ST7679, ST5837 and ST6320 (S1 Table). The *K. quasipneumoniae* (*n *= 11) were identified as having novel MLST profiles and were assigned ST5682 using the MLST scheme for *K. pneumoniae* as a foundation for differentiation (S2 Table). The most frequently recovered STs from dairy manure were ST278 (6 samples), ST183 (4 samples) and ST219 (4 samples) (Table 3), all of which were isolated from the largest farm of the study (Fig 1A). Many STs were only identified once (Table 3). Two *K. pneumoniae* STs, ST2159 and ST3369, were found on more than one farm (Fig 1A). None of the STs found in turkey feces or dog urine samples overlapped with those found in dairy manure (Fig 1D). The STs recovered from turkey feces included ST45 (*n* = 4), ST1564 (*n* = 2), ST215 (*n* = 1) and ST147 (*n* = 1). The two isolates from dog urine samples were both ST711 and were recovered from urine samples taken at different times from the same patient diagnosed with a UTI. It is unknown whether the UTI was due to a persistent infection or a re-infection with the same strain.

Of the STs found in our study, seven (ST107, ST1966, ST34, ST37, ST405, ST45 and ST711) were also found in the human clinical isolate database (National Center for Biotechnology Information (NCBI) BioProject PRJNA717739 [50]). Of these 7 human-associated isolates, four ST groups (ST37, ST405, ST45, and ST711) had similar ESC-resistance profiles to our *K. pneumoniae* samples from dairy manure and were resistant to ceftazidime, ceftriaxone and cefepime. From the human clinical isolates database, ST37 was from a urine sample in Asia in 2019, ST45 was from a respiratory sample in North America in 2013, ST405 was from a respiratory sample in South America in 2013, and ST711 was from a wound sample in Africa in 2019 [50].

**Fig 2:**
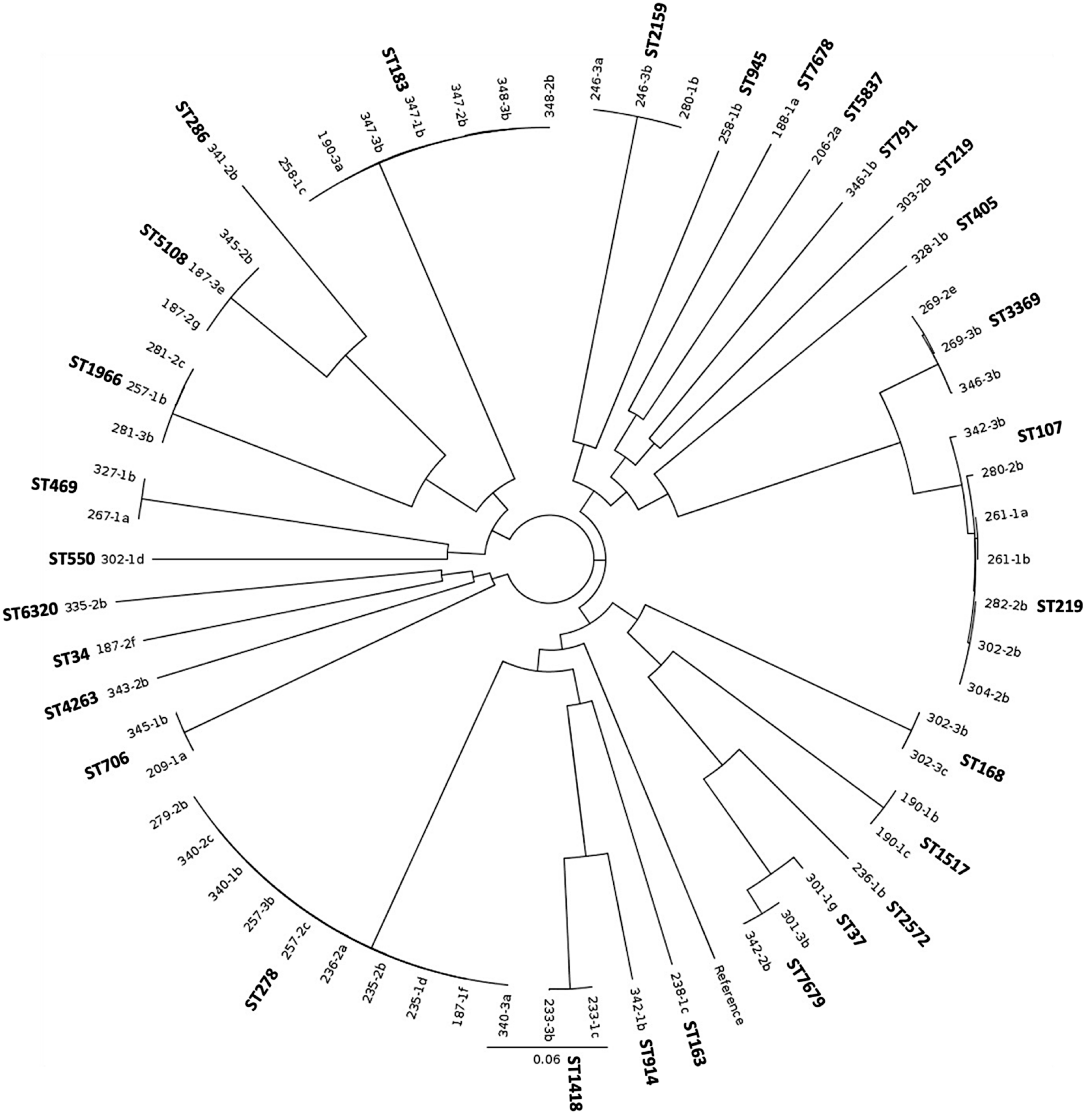
Single nucleotide polymorphism (SNP) maximum likelihood phylogenetic tree using 128,910 core SNP sites from *K. pneumoniae* isolates (*n* = 63) carrying *bla*_CTX-M_ in reference to *K. pneumoniae* type strain ATCC 13883 (GenBank Accession #JOOW00000000.1). Isolate identifications are comprised of the sample number, followed by replicate number and colony ID. Therefore, isolates with the same three digits are from the same sample and possibly clones.

The genomic similarities of isolates from all three animal sources were visualized using cgMLST Minimum Spanning Trees (MSTs) to identify any strain clustering among or between sources (Fig 1). Additionally, *Klebsiella spp*. isolates from dairy manure were further analyzed using the more discriminatory core SNP analysis to better understand distribution and persistence throughout manure treatments (Fig 2). Clusters of isolates were found to originate from several samples and therefore are not likely to be replicates. Clusters in both trees are supportive of one another. As mentioned above, only two clusters of isolates were found on more than one farm (Fig 1A) corresponding with ST2159 and ST3369. The ST2159 cluster includes three *bla*_CTX-M-15_-carrying isolates that are closely related and had no core SNP differences identified (246-3a, 246-3b, and 280-1b in Fig 2A). The ST3369 cluster includes two highly related isolates carrying *bla*_CTX-M-1_ (269-2e and 269-3b which have no SNP differences) from the same farm and sample, and one slightly different isolate (346-3b, 164 SNP differences from the other two isolates), which is from another farm and carried *bla*_CTX-M-32_ (Figs 1C and 2).

Two dairy manure isolates (ST219) carried *bla*_CTX-15_ on the chromosome and are linked by their source (Figs 1A and 1B), which was also the case for the two dog isolates (ST711). The two isolates from dairy manure (302-2b and 304-2b) (ST219, Fig 2) were from the same farm and sampling date, but different manure treatment stages. They had no core genome SNPs and had the same AMR gene profile. IS*Ecp1* insertion sequences were located upstream of the *bla*_CTX-M-15_ on the four chromosomes. For the dairy isolates, insertion had occurred within a conserved region of the chromosome and interrupted an aldose-1-epimerase gene. For the dog isolates, the insertion was instead in a different region with multiple IS elements and resistance genes, suggesting high plasticity.

### Persistence through dairy manure treatments

Using core genome SNP analysis on all sequenced *K. pneumoniae* isolates from dairy manure, excluding the eleven *K. quasipneumoniae*, nine clusters are evident (Fig 2). Of these, five [ST278 (0–31 SNPs, ST183 (0–31 SNPs), ST1966 (30–81 SNPs), ST706 (51 SNPs) and ST469 (20 SNPs)] contain closely related isolates which were recovered from different treatment stages and sampling dates. Two of the nine clusters [ST7679 (10 SNPs) and ST1966 (51–81 SNPs)] contain closely related isolates recovered from both the input and output stages of manure treatment from the same farm. Three identical ST5108 isolates were recovered from raw manure at different sampling periods but not found at other treatment stages. Additionally, closely related strains ST107 (*n* = 2) and ST219 (*n* = 5), were recovered from various stages of the treatment process (Fig 2). Within this branch, two ST219 (261-1a and 261-1b; 0 SNPs), likely to be the same clone, were recovered from heat treated compost in March, and were distinguishable by 288 SNPs from the other three ST219 isolates recovered from digestate with solids (302-2b in June) and dewatered manure (282-2b in April and 304-2b in June).

### Plasmid diversity

A subset of 35 isolates were sequenced with both long and short reads to enable ESC-resistance plasmid assembly (Table 4). Plasmids in isolates from dairy manure carried *bla*_CTX-M-15_ on IncY (*n* = 8), IncFII (*n* = 10), IncFIB(K) (*n* = 2), cointegrated IncR/IncFIA/Col (*n* = 2), IncFIB/IncHI1 (*n* = 1), and IncFIB(K)/IncFII (*n* = 1) replicons. Two of the isolates harbouring IncY replicons carrying *bla*_CTX-M-15_ were *K. quasipneumoniae*. Plasmids carrying *bla*_CTX-M-15_ from turkey samples were IncFIB(K)/IncFII (*n* = 6) or IncFIB(K) (*n* = 2). Two *K. pneumoniae* isolates from dairy manure carried *bla*_CTX-M-1_ on IncI1/ST3 plasmids and one isolate carried a *bla*_CTX-M-32_ on an IncN plasmid.

**Table 4:** Characteristics of *bla*_CTX-M_ plasmids of *K. pneumoniae* and *K. quasipneumoniae* isolated from dairy cattle (DC) manure on dairy farms or turkey (T) feces from Canadian sources.

Plasmid					Isolate		Source		
Replicon Type	Plasmid size (bp)	CTX-M	Additional AMR genes on CTX-M plasmid	pMLST	ID	Strain MLST	Animal	Stage*	Sample Date
IncFIB/HI1	309, 626	15	*bla*_*TEM-1*_ *, dfrA1*	F-:A-:B-	335-2b	ST6320	DC	Input	Aug/19
IncFIB(K)	158, 456	15	*aph(3’‘)-Ib, aph(6)-Id, bla*_TEM-1_ *, dfrA14, qnrB1, sul2*	F-:A-:B-	17.3	ST1564	T	N/A	Mar/17
	158, 458	15	*aph(3’‘)-Ib, aph(6)-Id, bla*_TEM-1_ *, dfrA14, qnrB1, sul2*	F-:A-:B-	17.1	ST1564	T	N/A	Mar/17
	205, 257	15	*aadA, aph(3’)-Ia, aph(3’‘)-Ib, aph(6)-Id, dfrA12, mph(A), qnrS1, sul1, sul2, tet(A)*	F-:A-:B-	327-1b	ST469	DC	Input	Aug/19
	211, 537	15	*aadA, aph(3’)-Ia, aph(3’‘)-Ib, aph(6)-Id, catA, dfrA12, mph(A), qnrS1, sul1, sul2, tet(A)*	F-:A-:B-	267-1a	ST469	DC	Inter.	Apr/19
IncFIB(K)/FII	172, 698	15	*aac(3)-IIa, aph(3’‘)-Ib, aph(6)-Id, bla*_OXA-1,_ *bla*_TEM-1_, *dfrA14, sul2*	K7:A-:B-	142.2	ST215	T	N/A	Aug/16
	194, 903	15	*aac(3)-IIa, aac(6’)-Ib-cr aph(3’‘)-Ib, aph(6)-Id, bla*_OXA-1_ *, bla*_TEM-1_ *, dfrA14, qnrB1, sul2, tet(A)*	K7:A-:B-	69.2	ST45	T	N/A	May/17
	194, 903	15	*aac(3)-IIa, aac(6’)-Ib-cr, aph(3’‘)-Ib, aph(6)-Id, bla*_OXA-1,_ *bla*_TEM-1_*, dfrA14, qnrB1, sul2, tet(A)*	K7:A-:B-	70.3	ST45	T	N/A	May/17
	194, 903	15	*aac(3)-IIa, aac(6’)-Ib-cr, aph(3’‘)-Ib, aph(6)-Id, bla*_OXA-1,_ *bla*_TEM-1_*, dfrA14, qnrB1, sul2, tet(A)*	K7:A-:B-	71.2	ST45	T	N/A	May/17
	194, 903	15	*aac(3)-IIa, aac(6’)-Ib-cr, aph(3’‘)-Ib, aph(6)-Id, bla*_OXA-1,_ *bla*_TEM-1_*, dfrA14, qnrB1, sul2, tet(A)*	K7:A-:B-	72.2	ST45	T	N/A	May/17
	222, 032	15	*aadA2, aac(6’)-Ib-cr, aph(3’)-Ia, bla*_OXA-1_ *, bla*_*TEM-1*_ *, dfrA12, mph(A), sul1, tet(A)*	K5:A-:B-	60.3	ST147	T	N/A	Jun/16
	237, 827	15	*aph(3’‘)-Ib, aph(6)-Id, bla*_TEM-1_ *, dfrA14, qnrB1, sul2, tet(A)*	K7:A-:B-	328-1b	ST405	DC	Inter.	Aug/19
IncFII	94, 092	15	*bla*_TEM-1_	K2:A-:B-	236-2a	ST278	DC	Inter	Feb/19
	94, 092	15	*bla*_TEM-1_	K2:A-:B-	340-1b	ST278	DC	Input	Aug/19
	94, 094	15	*bla*_TEM-1_	K2:A-:B-	235-2b	ST278	DC	Input	Feb/19
	94, 102	15	*bla*_TEM-1_	K2:A-:B-	187-1f	ST278	DC	Input	Dec/18
	94, 401	15	*bla*_TEM-1_	K2:A-:B-	261-1b	ST219	DC	Output	Mar/19
	94, 418	15	*bla*_TEM-1_	K2:A-:B-	187-2g	ST5108	DC	Input	Dec/18
	94, 426	15	*bla*_TEM-1_	K2:A-:B-	345-1b	ST706	DC	Input	Oct/19
	95, 483	15	*bla*_TEM-1_	K2:A-:B-	348-2b	ST183	DC	Inter.	Oct/19
	95, 484	15	*bla*_TEM-1_	K2:A-:B-	347-2b**	ST183	DC	Output	Oct/19
	95, 754	15	*bla*_TEM-1_	K2:A-:B-	342-1b	ST914	DC	Output	Aug/19
IncI1	104, 823	1	*sul2*	3	269-3b**	ST3369	DC	Inter.	Apr/19
	109, 290	1	*sul2, tet(A)*	3	269-2e**	ST3369	DC	Inter.	Apr/19
IncR/FIA	97, 075	15	*aac(3)-IIa, aph(3’‘)-Ib, aph(6)-Id, bla*_TEM-1_, *catA, dfrA14, sul2, tet(D)*	F-:A13:B-	233-1c	ST1418	DC	Output	Feb/19
	97, 408	15	*aac(3)-IIa, aph(3’‘)-Ib, aph(6)-Id, bla*_TEM-1_, *catA, dfrA14, sul2, tet(D)*	F-:A13:B-	233-3b	ST1418	DC	Output	Feb/19
IncY	88, 268	15	*dfrA14, qnrS1, tet(A)*		302-3b	ST168	DC	Inter.	Jun/19
	92, 997	15	*aph(3’‘)-Ib, aph(6)-Id, bla*_TEM-1_, *dfrA14, qnrS1, sul2*		**301-1c**	ST5682	DC	Input	Jun/19
	98, 474	15	*aph(3’‘)-Ib, aph(6)-Id, bla*_TEM-1_, *dfrA14, qnrS1, sul2, tet(A)*		341-2b	ST286	DC	Inter.	Aug/19
	98, 485	15	*aph(3’‘)-Ib, aph(6)-Id, bla*_TEM-1_, *dfrA14, qnrS1, sul2, tet(A)*		**279-3b**	ST5682	DC	Input	Apr/19
	98, 488	15	*aph(3’‘)-Ib, aph(6)-Id, bla*_TEM-1_ *, dfrA14, qnrS1, sul2, tet(A)*		343-2b	ST4263	DC	Inter.	Aug/19
	98, 488	15	*aph(3’‘)-Ib, aph(6)-Id, bla*_TEM-1_, *dfrA14, qnrS1, sul2, tet(A)*		281-2c	ST1966	DC	Output	Apr/19
	99, 264	15	*aph(3’‘)-Ib, aph(6)-Id, bla*_TEM-1_, *dfrA14, qnrS1, sul2, tet(A)*		246-3a	ST2159	DC	Output	Mar/19
	100, 397	15	*aph(3’‘)-Ib, aph(6)-Id, bla*_TEM-1_, *dfrA14, qnrS1, sul2, tet(A)*		238-1c	ST163	DC	Dew	Feb/19
IncN	37, 600	32	None	1	346-3b	ST3369	DC	Inter.	Oct/19

*Input refers to raw manure, output refers to either environmentally applied manure or dried solids which are used for bedding, and inter. refers to any intermediate stages between raw and output. Manure stages are only applicable for isolates recovered from dairy manure.

**Isolates used as donors for conjugation assays to *E. coli* recipients.

Bolded isolates are *K. quasipneumoniae* subsp. *quasipneumoniae.*Source acronyms are Dairy Cattle (DC) and Turkey (T).

The IncY replicons carrying *bla*_CTX-M-15_ (*n* = 8) were all from the same farm except for 246-3a. These IncY replicons were recovered from seven different STs (Table 4), and the samples they originated from spanned the entire manure treatment pipeline. However, the two isolates from raw input manure were both *K. quasipneumoniae* (301-1c and 279-3b). Despite being harbored by different hosts and on two separate farms, the IncY replicons were practically identical, with minor variations in copy number and location of insertion sequences (S1A Fig, S1B Fig). The IncY replicons identified in this study were annotated as conjugative plasmids, unlike many other documented IncY elements that have been classified as prophages or phage-plasmids [51], and they carried an extensive AMR region, including genes conferring resistance to tetracycline, sulfonamides, streptomycin, and aminopenicillins (S1 C Fig). Nearly identical IncY replicons from *E. coli* (MW077912.1; 99.99% nucleotide identify to the full length) were identified in the NCBI database and highly similar elements were also identified in a *Klebsiella pneumoniae* (CP132612.1; 100% identity, 91% coverage) and several *Salmonella enterica* isolates (e.g., CP173313.1; 100% identity, 94% coverage), Notably, a highly similar IncY- *bla*_CTX-M-15_ replicon was also found in an *E. coli* isolate from the same farm in our previous study [23], suggesting recent transfer between species. Conversely, an IncY-*bla*_CTX-M-55_ from another *E. coli* in that study did not carry conjugation genes, highlighting the functional diversity within this family of mobile elements.

**Fig 3:**
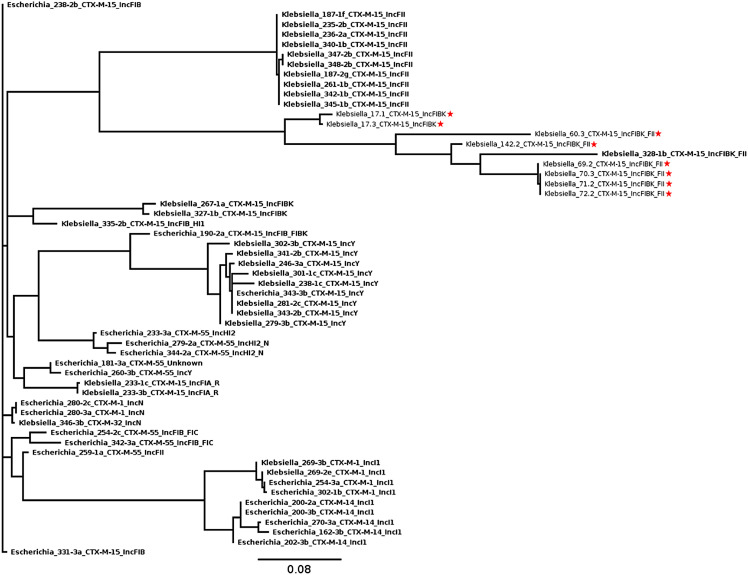
Phenetic tree based on presence and absence of 2,064 genes, including core genes (*n* = 330) and cloud genes (*n*  =1734), among plasmids carrying *bla*_CTX-M_ in *K. pneumoniae* and *E. coli* from dairy manure (boldface) and turkey feces (red stars). All isolates were recovered from sources in Ontario, Canada with the exception of isolates from turkey feces in British Columbia (17.1 and 17.3) and Quebec (69.2, 70.3, 71.2, 72.2 and 142.2).

**Fig 4:**
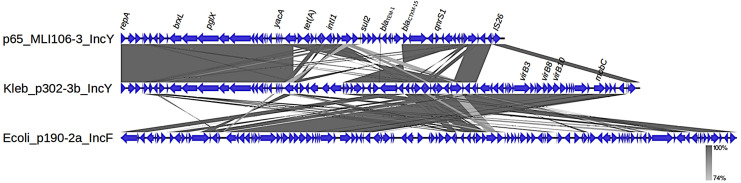
Alignment of one representative *bla*_CTX-M-15_ IncY plasmid from the dairy isolates aligned to a *bla*_CTX-M-15_ IncF plasmid from *E. coli* in the same samples and a non-conjugative *bla*_CTX-M-15_ IncY plasmid (CP117017.1). The IncY plasmid from our study is highly similar to the non-conjugative IncY from the replication genes until the antibiotic resistance region and then contains conjugation genes with high similarity to the IncFIB plasmid.

Assessment of plasmid similarity using the presence and absence of genes shows a good correlation between clusters and replicon types among isolates recovered from dairy cattle and turkey, and also with ESC-resistance plasmids identified in *E. coli* from the same dairy manure samples [23] (Fig 3). IncF plasmids were dominant and generally clustered according to their replicon subtypes, with some exceptions, which likely result from the high heterogenicity in this family of plasmids. Plasmids from the turkey samples remained distinct from those of dairy manure, with the exception of 328-1b, which was the only IncFIB(K)/FII isolated from dairy manure. Notably, one IncFIB plasmid was found at the root of the branch containing all of the IncY replicons. An alignment of these plasmids revealed that the conjugation genes associated with the IncY plasmids had a high similarity to this IncFIB plasmid. Further alignment with a non-conjugative IncY plasmid (CP117017.1) confirmed homology from the replication gene to the antibiotic resistance followed by the addition of the conjugation module in our plasmids (Fig 4)

The IncFII-CTX-M-15 plasmids were among the most frequent ESC-resistance replicons of the plasmids recovered from *Klebsiella spp.* in this study (n = 10). They were all carried by isolates recovered from farm seven, spanning the whole one-year sampling period, from December 2018 to October 2019. Based on core gene SNP analysis, these plasmids formed two main subclusters, with the exception of 261-1b (ST219) which was recovered in heat-treated compost (S2A Fig). Isolate 261-1b had 30–32 SNPs compared to the other isolates, which differed amongst themselves by only 0–2 SNPs. Clustering did not show any clear correlation with time or treatment stage. Few rearrangements were identified among the IncFII plasmids (S2B Fig), and these were not specifically associated with the AMR genes present in these plasmids (S2C Fig). One IncFII plasmid carrying *bla*_CTX-M-55_ was identified in *E. coli* from the same sources; however, it was structurally different and unrelated to the IncFII/*bla*_CTX-M-15_ plasmids from *Klebsiella spp*. (S3 Fig and Fig 3).

**Fig 5:**
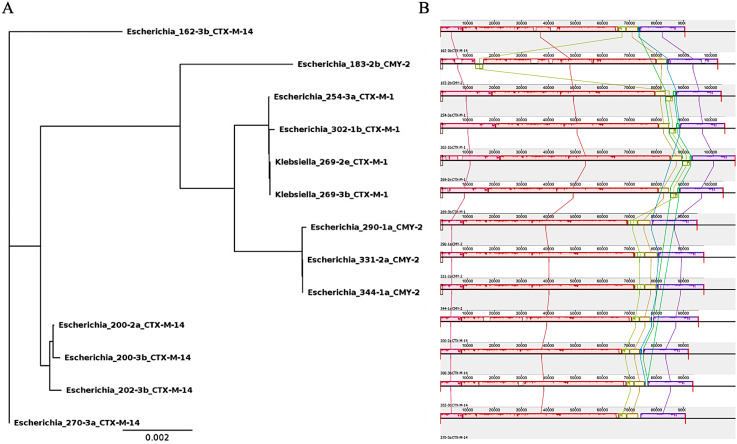
Comparison of IncI1 plasmids (*n* = 13) from both *K. pneumoniae* and *E. coli* carrying *bla*_CTX-M_ or *bla*_CMY_. A) Maximum likelihood-based on SNPs among 55,250 nucleotides among core plasmid loci and B) Mauve whole genome alignments of the whole plasmids.

IncI1 plasmids were also compared using core SNP analysis (62 genes; 0–649 SNPs in pairwise comparisons) and Mauve alignments (Fig 5). The two plasmids from *K. pneumoniae* clustered with two *E. coli* plasmids and all four were IncI1/ST3 plasmids carrying *bla*_CTX-M-1._ These IncI1-CTX-M-1 plasmids were not identical, but very similar both in their core gene sequences with 0 SNPs between the *K. pneumoniae* and 10 SNPs between the *E. coli* (Fig 5). Three of these were recovered from the same farm at different time periods but in samples from the same treatment stage (*K. pneumoniae* 269-2e, 269-3b and *E. coli* 254-3a). They were more similar to one another (0–3 SNPs) than to the plasmid recovered from an isolate from the other farm (10 SNPs). The IncI1-CTX-M-1 plasmid cluster was clearly distinct from the more diverse IncI-CMY-2 and IncI1-CTX-M-14 plasmids (Fig 5). Between *K. pneumoniae* and *E. coli* there were 3–593 SNPs.

### Plasmid mobility

Both IncI1 and IncFII plasmids carrying *bla*_CTX-M-1_ and *bla*_CTX-M-15_, respectively, were transferred when using *K. pneumoniae* strains as donors and an *E. coli* recipient, with higher conjugation efficiency at 37˚C for all three donors compared to 30˚C (Table 5). Among these three donors, IncI1 plasmids transferred at approximately 100x higher rates than the IncFII plasmid. In contrast, when *E. coli* donors were used with a *K. pneumoniae* recipient, no transfer of *bla*_CTX-M_ plasmids was detected in either broth or surface mating. Conjugation of IncY plasmids was also confirmed between *E. coli* strains, as well as from *Klebsiella* to *E. coli*, however transfer was found to occur at low frequency (2.67x10^-6^) and was only confirmed for surface mating at 37 ˚C.

**Table 5:** Transfer frequencies of *bla*_CTX-M_ plasmids from *K. pneumoniae* donor strains to *E. coli* CV601*gfp*Kan^R^Rif^R^ recipient strain using broth mating.

Donor	Replicon Type	CTX-M	Conjugation Temp.	Transfer Frequency
269-2e	IncI1/ST3	1	30˚C	6.14x10^-3^
			37˚C	3.2x10^-2^
269-3b	IncI1/ST3	1	30˚C	2.81x10^-2^
			37˚C	4.0x10^-2^
347-2b	IncFII	15	30˚C	2.99x10^-5^
			37˚C	3.47x10^-4^

## Discussion

ESC-resistance is a One Health issue, yet very little is known about the epidemiology of ESC-R *K. pneumoniae* in animals in Canada, despite their importance in both animal and human clinical infections [10,52]. There is potential for transmission of ESC-resistant strains and their AMR determinants between humans and animals, through close proximity or consumption of contaminated food via crop amendment or meat products [16,53–56]. Therefore, this study sought to further characterize ESC-R *K. pneumoniae* isolates from dairy manure in terms of their strain diversity, antimicrobial resistance, and potential contribution to the transmission dynamics of ESC-R plasmids in manure.

A large proportion (86.5%) of *Klebsiella pneumoniae* isolates carrying *bla*_CMY_ or *bla*_CTX-M_ were MDR, regardless of animal source. Similarly, in our parallel study, there were 79% MDR isolates among ESC-resistant *E. coli* from the same manure samples [23]. The majority of resistances observed in dairy manure isolates were to TET, STR, SXT and SUL, which are some of the most commonly prescribed antimicrobials in veterinary medicine, including for dairy cattle [57]. This followed again the same trend as in *E. coli* [23]. Twenty-six percent of the *Klebsiella spp*. from dairy cattle showed an intermediate susceptibility to CIP (1% in *E. coli*), but full resistances to CIP, CHL, KAN and GEN were low and in similar ranges in *Klebsiella* spp. and *E. coli* [23]. All ESC-R *Klebsiella* spp. isolates were susceptible to ETP and no resistances were identified to other critically important antimicrobials for human medicine.

The majority of isolates from this study were from a single farm and there were ESC-resistant isolates recovered at every manure treatment stage. In contrast, recovery on the other farms was sporadic and no ESC-resistant *K. pneumoniae* were recovered at all from two of the six farms investigated [22]. This suggests that contrary to the situation with *E. coli*, *K. pneumoniae* carrying *bla*_CTX-M_ may not be endemic to all farms or are more challenging to detect using our current methods. Overall, due to the low recovery rate of *K. pneumoniae* and *K. quasipneumoniae* isolates, it is difficult to assess the effect manure treatments may have on their prevalence and more extensive quantitative analyses would be needed for this.

In keeping with the high *K. pneumoniae* strain diversity observed in dairy manure (30 STs), only two STs were found on more than one farm, ST3369 and ST2159. Both were found on the largest farm investigated (farm seven) and one additional farm. Additionally, ST7679 and ST1966 were recovered at different treatment stages, ranging from raw to environmentally applied manure and at different sampling times. In both cases, isolates were closely related but not identical suggesting that these strains may remain in the system over extended periods of time. Further studies would be needed to assess whether these strains may possess adaptive features for the manure environment. In addition, eleven *K. quasipneumoniae* isolates were recovered from farm seven at three different treatment stages, with the majority found in the raw manure. This may indicate recurrent entrance into the system and limited success at surviving the conditions encountered during manure treatment. Comparing the dairy manure strains with other animal sources, there was extensive strain and plasmid diversity within animal sources and no overlaps at the clonal level between different animal sources in this study. *K. pneumoniae* strains frequently associated with human infections globally include ST11, ST13, ST14, ST15, ST37, ST147, ST258, ST307, ST405 or ST512 [1,58–62]. Of these, only two were identified in our study (ST37 (raw manure) and ST405 (anaerobically digested manure)). ST37 is suspected to have spread globally through importation and trade and is considered a highly virulent lineage [63]. The low frequency of these *K. pneumoniae* strains in dairy cattle manure is reassuring but requires future surveillance.

Contrary to the situation in *E. coli* [23] only a small number of the ESC-resistant isolates carried a *bla*_CMY_ gene and most had a *bla*_CTX-M_ gene. Three *bla*_CTX-M_ variants were identified and all belonged to enzyme group one [64]. The most common variant was *bla*_CTX-M-15_, which is known to be widespread globally in *K. pneumoniae* from both human and veterinary clinical infections [65–69]. Similar to what has been observed in *E. coli* from the same manure samples, the *bla*_CTX-M-15_ was often carried on the same plasmid as *bla*_TEM-1_ [23,49]. The overwhelming prevalence of *bla*_CTX-M-15_ and the infrequent recovery of *bla*_CTX-M-1_ and *bla*_CTX-M-32_ suggests a low diversity of *bla*_CTX-M_ variants in *K. pneumoniae* from dairy manure. This seems to contrast with the situation in *E. coli* in which seven *bla*_CTX-M_ variants were identified in our parallel study of these same samples, and *bla*_CTX-M-15_ was found in only 55% of cases [23]. *K. pneumoniae* intrinsically carry *bla*_SHV_ variants, which are typically the non-ESBL variants *bla*_SHV-1_ and *bla*_SHV-11_, [70,71]. However, in rare cases they can carry *bla*_SHV-27_, which is variant documented to have an ESBL phenotype. Four isolates of two unrelated STs from farm seven were identified carrying *bla*_SHV-27_ in this study, though the ESBL phenotype of this variant could not be confirmed due to co-occurrence with CTX-M genes. The prevalence of ESBL SHV variants appears to be low currently, but monitoring may be warranted.

The majority of *bla*_CTX-M_ genes were carried on plasmids. Only two isolates from dairy manure and the two dog isolates carried their *bla*_CTX-M-15_ on the chromosome. In each case, the chromosomal integration was likely due to mobilization through the action of IS*Ecp*1. This insertion sequence is frequently associated with the mobilization of *bla*_CTX-M-15_ [72] and our findings are therefore not entirely surprising.

The majority of plasmids carrying *bla*_CTX-M-15_ were replicon type IncF and very diverse (Fig 3). This is similar to what is found in *E. coli* from the same manure samples, with highly diverse IncF plasmids carrying *bla*_CTX-M-15_ and *bla*_CTX-M-55_ [23]. Very similar IncFII plasmids were found in multiple genetically unrelated *Klebsiella spp.* strains in one of the farms investigated (Table 4; Fig S2), suggesting that these plasmids may be highly mobile within *Klebsiella* strains in the farm and manure treatment environments. In contrast, only one IncFII plasmid (carrying *bla*_CTX-M-55_) was identified in *E. coli* from the same dairy manure samples [23]. It was structurally different than the IncFII plasmids carrying *bla*_CTX-M-15_ in *Klebsiella spp*. This may indicate differences in the dynamics among IncF subtypes for *Klebsiella spp.* in general, or specifically for *Klebsiella* strains within manure. *E. coli* and *Klebsiella spp.* commonly carry different IncFII replicon alleles [73], which may be a factor in the dissemination dynamics observed.

Only two genetically related but not identical isolates from the same farm (Fig 1) were found to carry *bla*_CTX-M-1_. It was located on an IncI1/ST3 “epidemic” plasmid [74] and a similar plasmid was also detected in *E. coli* from the same manure samples [23]. The IncI1 plasmid tested transferred at rates hundreds to thousands time higher than the IncFII plasmids. This plasmid may have recently been introduced to the dairy cattle environment in Canada or Ontario, as suggested by its low frequency in both *K. pneumoniae* and *E. coli.* Its high level of transferability and common presence in other commodities such as poultry in Canada [46] suggest a need for further monitoring of its spread in dairy cattle and manure. Conjugation rates for both IncI1 and IncFII plasmids from *K. pneumoniae* to *E. coli* were higher at increased mesophilic temperatures, 37˚C, similar to those used on the dairy farms during anaerobic digestion. Conjugative transfer was successfully achieved between *K. pneumoniae* and *E. coli*, but not between *E. coli* and *K. pneumoniae* for any of the plasmid types tested. However, only one *K. pneumoniae* strain was tested as a recipient and the lack of transfer may be related to characteristics specific to this strain rather than to a general inability to transfer from *E. coli* to *K. pneumoniae*.

The high similarity of IncY replicons supported the transmission of *bla*_CTX-M-15_ plasmids between *E. coli*, *K. pneumoniae* and *K. quasipneumoniae*., which was further corroborated by our conjugation experiments demonstrating transfer from *K. pneumoniae* to *E. coli*. Due to the recent description of *K. quasipneumoniae*, little is known about its role in ESC-resistance and transmission [75]. However, *K. quasipneumonaie* carrying *bla*_CTX-M-2_ among other AMR genes, has been recovered in human sewage in Japan, and was capable of transferring *bla*_CTX-M_-harbouring IncA/C plasmids to *E. coli* [76]. Our findings may indicate that *K. quasipneumoniae* is also involved in the persistence and transmission of ESC-resistance plasmids in the manure environment. This information further stresses the diversity of potential hosts for resistance plasmids. The occurrence of conjugative IncY plasmids carrying multiple resistance genes highlights the need for more research into the role of phage-plasmids and similar MGEs in the evolution of antimicrobial resistance [51,77].

## Conclusions

Although diverse strains were identified, ESC-resistant *K. pneumoniae* were not recovered from every farm, and only sporadically in the positive ones. Thus, these bacteria may play a small role in the broad dissemination of ESC-resistance. However, under specific circumstances, as found in one of the farms under investigation from which the majority of isolates originated, *K. pneumoniae* could act as reservoirs of transmission within local bacterial populations. The successful transfer of *bla*_CTX-M_ plasmids between *K. pneumoniae* and *E. coli* as well as the demonstration *in silico* of plasmid similarities between *K. pneumoniae* and *K. quasipneumoniae* further supports the need to investigate not only *E. coli*, but also other members of the Enterobacterales, in attempts to better understand the dynamics and epidemiology of ESC-resistance in manure.

## Supporting information

S1 Table Temporary multi-locus sequence typing identifications for *K. pneumoniae* isolates with novel alleles or novel allelic configurations. (DOCX)

S2 Table Multi-locus sequence typing (MLST) identifications for *K. quasipneumoniae* isolates based on the MLST scheme for *K. pneumoniae* to enable strain differentiation. (DOCX)

S1 Fig Phylogenetic maximum likelihood SNP analysis (A) on IncY-*bla*_CTX-M-15_ plasmids using core genes (*n *= 73) with mauve alignments (B) and annotated plasmid segment carrying AMR genes (C). Plasmids were from *K. pneumoniae* and *K. quasipneumoniae* recovered from dairy manure. Plasmid names are colour coded (A) based on manure process in which they were recovered, raw manure (red), digestate with solids (green), digestate without solids (orange), dewatered (blue). All plasmids are harboured in isolates from farm seven, except for 246-3a.
(DOCX)

S2 Fig Phylogenetic maximum likelihood SNP analysis (A) on IncFII-*bla*_CTX-M-15_ plasmids using core genes (*n* = 121) with mauve alignments (B) and annotated plasmid segment carrying AMR genes (C). All plasmids are harboured in *K. pneumoniae* recovered from dairy manure on farm seven. Nodes are colour coded (A) based on manure process in which they were recovered, raw manure (red), digestate with solids (orange), digestate without solids (green), dewatered (blue) and heat-treated compost (purple).
(DOCX)

S3 Fig Comparison of IncFII plasmids from one *E. coli* (259-1a) and *K. pneumoniae* (*n* = 10) recovered from dairy manure using mauve alignments. (DOCX)
