# Supplementary material for: Characterization of extended-spectrum cephalosporin-resistant Klebsiella recovered from dairy manure in Southern Ontario, Canada
Source: PLoS One. 2026 Jan 9;21(1):e0336012. doi: 10.1371/journal.pone.0336012 (PMC12788680; doi:10.1371/journal.pone.0336012)
Supplement: S1 Table — (DOCX) [file pone.0336012.s001.docx]

Table S1 Temporary multi-locus sequence typing identifications for *K. pneumoniae* isolates with novel alleles or novel allelic configurations.

| Novel MLST ID | Novel Allele | Novel Allelic Configuration | MLST alleles for *K. pneumoniae* | | | | | | | Isolate ID |
| --- | --- | --- | --- | --- | --- | --- | --- | --- | --- | --- |
|  |  |  | *gapA* | *infB* | *mdh* | *pgi* | *phoE* | *rpoB* | *tonB* |  |
| ST7678 |  | yes | 2 | 1 | 2 | 1 | 1 | 4 | 9 | 188-1a |
| ST5837 |  | no | 3 | 1 | 1 | 3 | 3 | 28 | 39 | 206-2a |
| ST7679 |  | yes | 2 | 9 | 2 | 1 | 13 | 1 | 100 | 301-3b, 342-2b |
| ST6320 | yes |  | 2 | 1 | 13 | 26 | 21 | 5 | 876 | 335-2b |
