# Supplementary material for: Characterization of extended-spectrum cephalosporin-resistant Klebsiella recovered from dairy manure in Southern Ontario, Canada
Source: PLoS One. 2026 Jan 9;21(1):e0336012. doi: 10.1371/journal.pone.0336012 (PMC12788680; doi:10.1371/journal.pone.0336012)
Supplement: S2 Table — (DOCX) [file pone.0336012.s002.docx]

**Table S2** Multi-locus sequence typing (MLST) identifications for *K. quasipneumoniae* isolates based on the MLST scheme for *K. pneumoniae* to enable strain differentiation.

| Isolate ID^1^ | MLST alleles for *K. pneumoniae* | | | | | | |
| --- | --- | --- | --- | --- | --- | --- | --- |
|  | *gapA* | *infB* | *mdh* | *pgi* | *phoE* | *rpoB* | *tonB* |
| ST5682 | SNP | 19 | SNP | 39 | SNP | 21 | 162 |
