# Supplementary material for: Characterization of extended-spectrum cephalosporin-resistant Klebsiella recovered from dairy manure in Southern Ontario, Canada
Source: PLoS One. 2026 Jan 9;21(1):e0336012. doi: 10.1371/journal.pone.0336012 (PMC12788680; doi:10.1371/journal.pone.0336012)
Supplement: S1 Fig — Plasmids were from K. pneumoniae and K. quasipneumoniae recovered from dairy manure. Plasmid names are colour coded (A) based on manure process in which they were recovered, raw manure (red), digestate with solids (green), digestate without solids (orange), dewatered (blue). All plasmids are harboured in isolates from farm seven, except for 246-3a. (DOCX) [file pone.0336012.s003.docx]

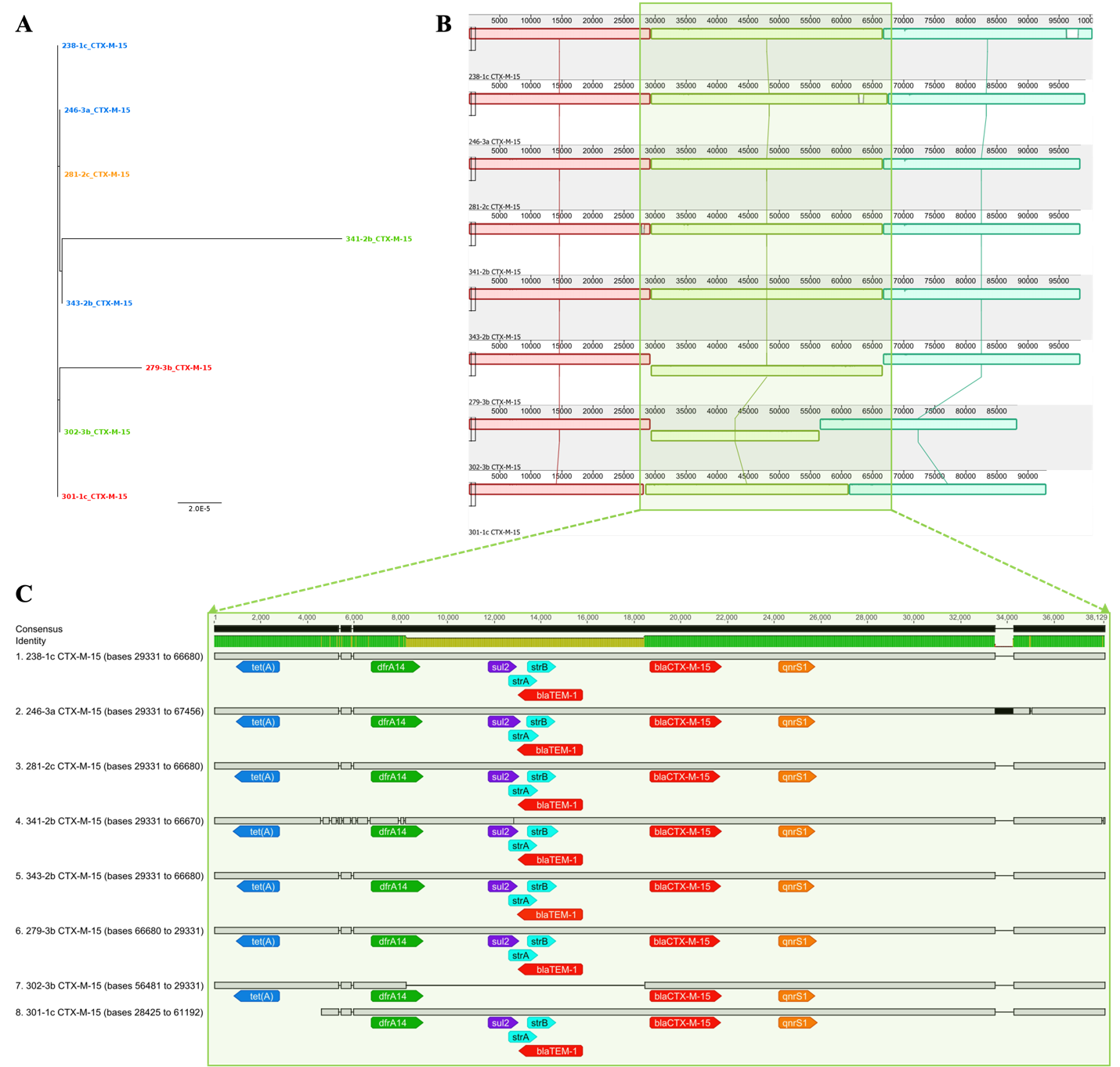


**Figure S1.** Phylogenetic maximum likelihood SNP analysis (A) on IncY-*bla*_CTX-M-15_ plasmids using core genes (*n*= 73) with mauve alignments (B) and annotated plasmid segment carrying AMR genes (C). Plasmids were from *K. pneumoniae* and *K. quasipneumoniae* recovered from dairy manure. Plasmid names are colour coded (A) based on manure process in which they were recovered, raw manure (red), digestate with solids (green), digestate without solids (orange), dewatered (blue). All plasmids are harboured in isolates from farm seven, except for 246-3a.
