# Supplementary material for: Characterization of extended-spectrum cephalosporin-resistant Klebsiella recovered from dairy manure in Southern Ontario, Canada
Source: PLoS One. 2026 Jan 9;21(1):e0336012. doi: 10.1371/journal.pone.0336012 (PMC12788680; doi:10.1371/journal.pone.0336012)
Supplement: S2 Fig — All plasmids are harboured in K. pneumoniae recovered from dairy manure on farm seven. Nodes are colour coded (A) based on manure process in which they were recovered, raw manure (red), digestate with solids (orange), digestate without solids (green), dewatered (blue) and heat-treated compost (purple). (DOCX) [file pone.0336012.s004.docx]

**Figure S2.** Phylogenetic maximum likelihood SNP analysis (A) on IncFII-*bla*_CTX-M-15_ plasmids using core genes (*n* = 121) with mauve alignments (B) and annotated plasmid segment carrying AMR genes (C). All Plasmids are harboured in *K. pneumoniae* recovered from dairy manure on farm seven. Nodes are colour coded (A) based on manure process in which they were recovered, raw manure (red), digestate with solids (orange), digestate without solids (green), dewatered (blue) and heat-treated compost (purple).
