# Supplementary material for: Characterization of extended-spectrum cephalosporin-resistant Klebsiella recovered from dairy manure in Southern Ontario, Canada
Source: PLoS One. 2026 Jan 9;21(1):e0336012. doi: 10.1371/journal.pone.0336012 (PMC12788680; doi:10.1371/journal.pone.0336012)
Supplement: S3 Fig — (DOCX) [file pone.0336012.s005.docx]

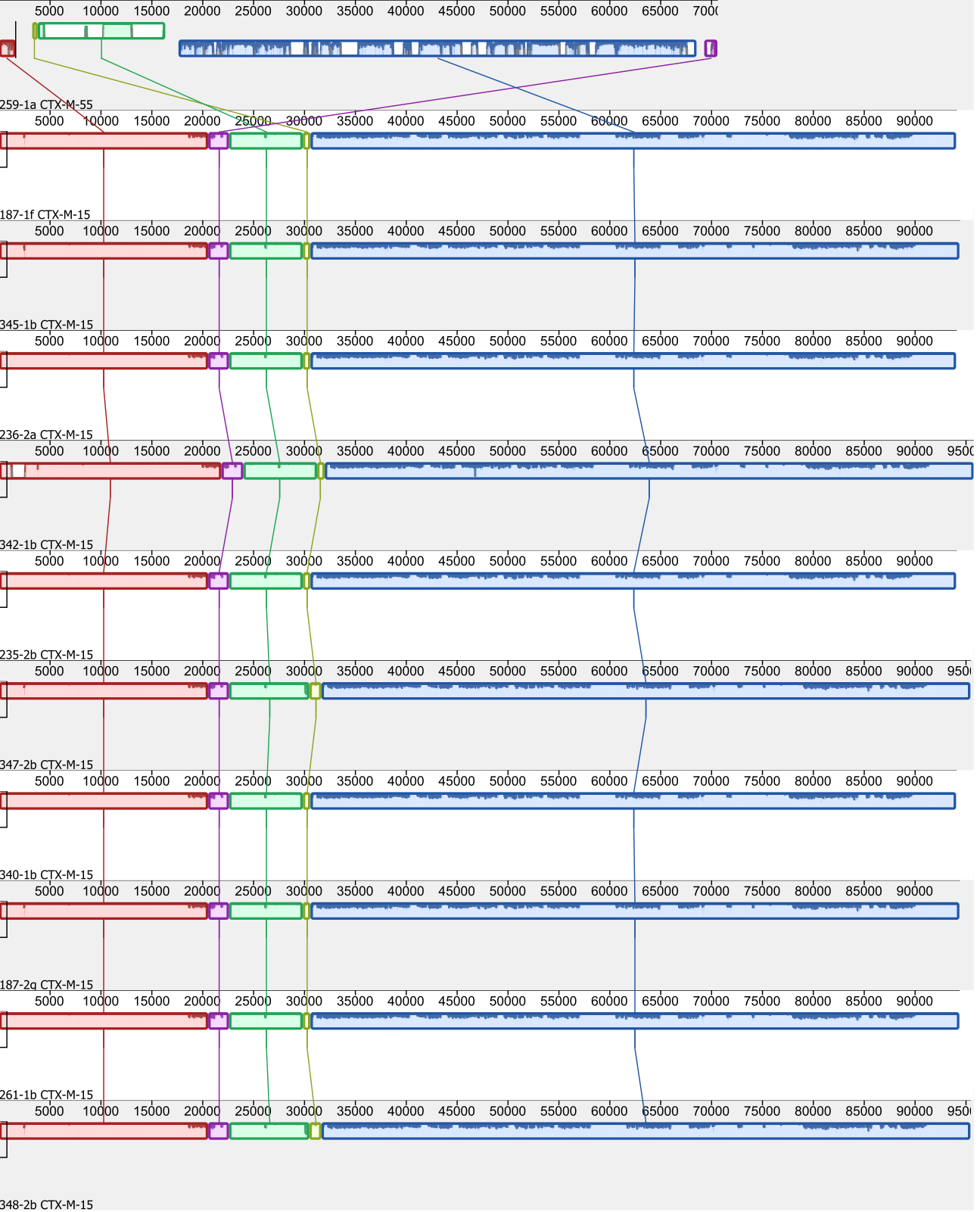


**Figure S3**. Comparison of IncFII plasmids from one *E. coli* (259-1a) and *K. pneumoniae* (*n* = 10) recovered from dairy manure using Mauve alignments.
